# Nuclear, Mitochondrial and Plastid Gene Phylogenies of *Dinophysis miles* (Dinophyceae): Evidence of Variable Types of Chloroplasts

**DOI:** 10.1371/journal.pone.0029398

**Published:** 2011-12-29

**Authors:** Dajun Qiu, Liangmin Huang, Sheng Liu, Senjie Lin

**Affiliations:** 1 Key Laboratory of Marine Bio-resources Sustainable Utilization, South China Sea Institute of Oceanology, Chinese Academy of Science, Guangzhou, China; 2 Tropical Marine Biological Research Station in Hainan, Chinese Academy of Sciences, Sanya, China; 3 Department of Marine Sciences, University of Connecticut, Groton, Connecticut, United States of America; University of Melbourne, Australia

## Abstract

The *Dinophysis* genus is an ecologically and evolutionarily important group of marine dinoflagellates, yet their molecular phylogenetic positions and ecological characteristics such as trophic modes remain poorly understood. Here, a population of *Dinophysis miles* var. *indica* was sampled from South China Sea in March 2010. Nuclear ribosomal RNA gene (rDNA) SSU, ITS1-5.8S-ITS2 and LSU, mitochondrial genes encoding cytochrome B (*cob*) and cytochrome C oxidase subunit I (*cox*1), and plastid rDNA SSU were PCR amplified and sequenced. Phylogenetic analyses based on *cob*, *cox*1, and the nuclear rRNA regions showed that *D. miles* was closely related to *D. tripos* and *D. caudata* while distinct from *D. acuminata*. Along with morphology the LSU and ITS1-5.8S-ITS2 molecular data confirmed that this population was *D. miles* var. *indica*. Furthermore, the result demonstrated that ITS1-5.8S-ITS2 fragment was the most effective region to distinguish *D. miles* from other *Dinophysis* species. Three distinct types of plastid rDNA sequences were detected, belonging to plastids of a cryptophyte, a haptophyte, and a cyanobacterium, respectively. This is the first documentation of three photosynthetic entities associated with a *Dinophysis* species. While the cyanobacterial sequence likely represented an ectosymbiont of the *D. miles* cells, the detection of the cryptophyte and haptophyte plastid sequences indicates that the natural assemblage of *D. miles* likely retain more than one type of plastids from its prey algae for temporary use in photosynthesis. The result, together with recent findings of plastid types in other *Dinophysis* species, suggests that more systematic research is required to understand the complex nutritional physiology of this genus of dinoflagellates.

## Introduction

The *Dinophysis* genus is an ecologically important group of dinoflagellates. *Dinophysis* spp. play dual roles in the marine ecosystems: as primary (photosynthetic) and secondary (heterotrophic) producers. Furthermore, many *Dinophysis* species are known to produce potent polyether toxins. For instance, *D. caudata* and *D. miles* have formed blooms and caused diarrhetic shellfish poisoning through accumulation of toxins in the green mussel [1]. Therefore, the genus *Dinophysis* is important in microbial food webs and for its potential influence on public health [2]. In addition, *Dinophysis* spp. have peculiar and unique morphologies that are not shared by any organisms outside the class of Dinophysiales, making this genus an interesting subject of evolutionary studies. However, until recently their phylogenetic position among dinoflagellates and their ecology such as trophic modes have remained poorly understood in most species due to the paucity of cultures or tools to study wild populations.

The genus *Dinophysis* has an obscure phylogenetic position among dinoflagellates. Using rRNA gene (rDNA) small subunit (SSU) and mitochondrial genes encoding cytochrome B (*cob*) and cytochrome C oxidase subunit I (*cox*1) and its mRNA editing patterns, a natural population of *D. acuminata* was placed phylogenetically between Gonyaulacales and Prorocentrales [3]. Recently, a sister kinship to *Phalacoma* was established for the genus *Dinophysis* [4], [5]. Dinophysioids have diverse trophic modes; some species are heterotrophic feeding on other algae [6], [7], whilst others have intracellular and extracellular cyanobionts and probably acquire carbon fixed by these symbionts. In *Histioneis* and *Ornithocercus*, the cyanobionts resides on the cingular lists [4], [8]–[11], whereas *Amphisolenia* [12], [13] and *Sinophysis canaliculata* cells [14] host the cyanobionts intracellularly. Typical *Dinophysis* spp. have been found to contain a plastid of cryptophyte origin [7], [15]–[17], in most cases *Teleaulax*-derived [2], although whether such uniformity in plastid acquisition is likely in other species and whether the plastids are kleptoplasts or permanent plastids have been debated [17]–[21]. Hackett *et al*. (2003) detected plastid rDNA sequences of a cryptophyte and a rhodophyte in *D. acuminata* and attributed the former to plastid and the latter to prey [22]. Meanwhile, *D. mitra* was found to harbor plastids of haptophyte origin [23].

The recent success in culturing *D. acuminata* [24] has greatly facilitated physiological, phylogenetic and molecular studies of the genus [25]–[27]. However, because the number of *Dinophysis* cultures is currently limited, work on many species still relies on natural populations. Work on natural populations not only broadens the range of species to be studied, but also can reveal in situ status of physiology and gene expression (e.g., [28]). A population of *D. acuminata* was isolated via flow cytometer from Narragansett Bay that enabled both the detection of mitochondrial mRNA editing in this species and its phylogenetic position based on nuclear rDNA SSU [3]. More phylogenetic studies have been conducted for natural populations from Florida embayments [4] and Indian Ocean [5]. rDNA LSU and SSU have been used to determine the relationship between the genera *Phalacroma* and *Dinophysis* [4]–[5], although their resolving power has yet to be demonstrated in some species in the *Dinophysis* genus. For instance, a study showed that rDNA LSU failed to distinguish *D. miles* from *D. tripos*, and *D. odiosa* [5]. To date, hardly any studies have been dedicated to *D. miles*, and the plastid type of this species remains undocumented. *D. miles* is recognized as variant *D. miles* var. *schroeteri* in Southeast Asia and *D. miles* var. *indica* in Indo-West Pacific [29], the latter widely distributed in the northeast area of South China Sea, such as Hainan island and Nansha islands waters [30]. In this study, we have investigated the phylogenetic position and plastid types of *D. mil*es var. *indica* from South China Sea.

## Materials and Methods

### Sample collection

A phytoplankton sample was collected at 18°11.5′N, 119°27′E (latitude, longitude) near Sanya in the South China Sea with a 55-µm mesh plankton net in March, 2010. The towed sample was transferred into a 500-mL plastic container and preserved with neutral Lugol's solution [31]. The sample was stored in the laboratory in the dark until analysis (within 3 months).

### Microscopic observations and cell sorting

Microscopic examination of the preserved phytoplankton sample revealed an abundant population of *D. miles*. The abundance of this species and other phytoplankton in the sample was determined using Sedgwick-Rafter chamber. Identification of the species was carried-out according to Steidinger (1997) and Wood (1963) [9] [32]. The abundance of this species in the natural environment was estimated by adjusting the cell concentration in the retrieved sample to the volume of water filtered in the net tow. Morphocytological features were examined both under Lugol's staining and after Lugol's stain was removed. To remove Lugol's stain, a subsample was centrifuged and supernatant discarded. The cell pellet was rinsed with 0.45-µm filtered seawater, followed by treatment with 10% (weight/volumn) sodium thiosulfate [33]. DNA was stained using SYBR Green I (35149A, Molecular probes, Invitrogen Corporation, Carlsbad, CA, USA) at 1∶10000 dilution at room temperature for 30 min [34]. DNA and pigment fluorescence was observed under an Olympus BX51 epifluorescence microscope. From the original Lugol's-preserved samples, colonies consisting of eight *D. miles* cells were isolated under the inverted microscope. The isolated cells were rinsed carefully with 0.45-µm filtered seawater for subsequent DNA extraction.

### DNA extraction, PCR, and gene sequencing

Four eight-cell *D. miles* colonies were resuspended in 0.5 mL DNA lysis buffer (0.1 M EDTA pH 8.0, 1% SDS, 200 µg mL^−1^ proteinase K) and incubated for 48 hours at 55°C. DNA extraction followed a previously reported protocol [35]. Briefly, after incubation, NaCl was added to achieve 0.7 M, and CTAB was added to the final concentration of 1.7%. The lysate was then extracted in chloroform. After centrifugation, the supernatant was removed and DNA further purified using Zymo DNA Clean and Concentrator kit (Zymo Research Corp., Orange, CA). At last, DNA was eluted in 32 µl Tris-HCl solution so that each µl contained DNA from about 1 cell of *D. miles*.

Using 1 µl of the extracted DNA as the template, PCR reactions were carried out using a pair of dinoflagellate-specific rDNA SSU primers [31], a pair of rDNA primers extended from internal transcribed spacer (ITS) to LSU regions [4], [36], [37], a pair of *cob* primers [3], a pair of *cox*1 primers [3], and a pair of plastid rDNA SSU primers [38]. The sequences of the primers were as shown in Table 1. PCR cycles consisted of one initial cycle of denaturation at 94°C for 3 min followed by 35 cycles of at 94°C for 30 sec, 56°C for 30 sec, and 72°C for 45 sec, followed by 10 min at 72°C for the final extension. PCR products were resolved on an agarose gel electrophoretically and the specific DNA band was excised. DNA was recovered and purified using a Zymo DNA column and sequenced directly using BigDye sequencing kit. For the plastid rDNA SSU, direct sequencing of the PCR product indicated the presence of different sequences. Therefore, the purified PCR product was ligated, cloned, and multiple clones were sequenced on both strands of the DNA.

**Table 1:** Primers used in the present study.

Primer name	Sequence (5′–3′)	References
Dino18SF1	AAGGGTTGTGTTYATTAGNTACARAAC	Lin *et al.*, 2006
18ScomR1	CACCTACGGAAACCTTGTTACGAC	Zhang *et al.*,2005
Dino1662 F	CCGATTGAGTGWTCCGGTGAATAA	Handy *et al.*, 2008
25R	CTTGGTCCGTGTTTCAAGAC	Yamaguchi *et al.*, 2005
Dinocob1F	ATGAAATCTCATTTACAWWCATATCCTTGTCC	Zhang *et al.*, 2008
Dinocob2R	CGAGCATAAGATAKAAACWTCTCTTGAGG	Zhang *et al.*, 2008
DinocoxF	AAAAATTGTAATCATAAACGCTTAGG	Zhang *et al.*, 2008
DinocoxR	TGTTGAGCCACCTATAGTAAACATTA	Zhang *et al.*, 2008
CYA361f	GGAATTTTCCGCAATGGG	Martin *et al.*, 2008
CYA785r	GACTACWGGGGTATCTAATCC	Martin *et al.*, 2008

### Phylogenetic analyses

DNA sequences were trimmed of primers and the two strands were merged. The assembled sequences were analyzed using Basic Local Search Tool (BLAST) against databases in GenBank to determine what organisms these rDNA sequences represented. Sequences showing significant similarity in BLAST to the sequences obtained in this study were retrieved from the databases. Phylogenies based on partial SSU, ITS1-5.8S-ITS2, partial LSU (D1-D2, 700-bp; [4]), *cob* (334-bp), and *cox*1 (840-bp) regions were used to investigate the phylogenetic position of *D. miles*. Phylogenetic trees were also inferred from plastid rDNA SSU to analyze the plastid type in *D. miles*. These datasets were separately aligned using ClustalX. The alignments were run through ModelTest to select the most appropriate evolutionary model. The selected General Time Reversible (GTR) model with gamma distribution was employed for Maximum Likelihood analysis using PhyML3.0 aLRT [39]. Categories of substitution rates were set at 4, and other parameters were estimated based on the datasets. The proportion of invariable sites and gamma shape parameter were 0.464 and 0.583, respectively for the SSU dataset, 0.127 and 1.296 for ITS, 0.185 and 0.689 for LSU, 0.098 and 1.130 for *cob*, 0.000 and 0.725 for *cox*1, and 0.214 and 0.360 for plastid SSU.

### Nucleotide sequence accession numbers

The sequences obtained in this study were deposited in GenBank under accession numbers JN982970-JN982975.

## Results

### Microscopic observations

**Figure 1:**
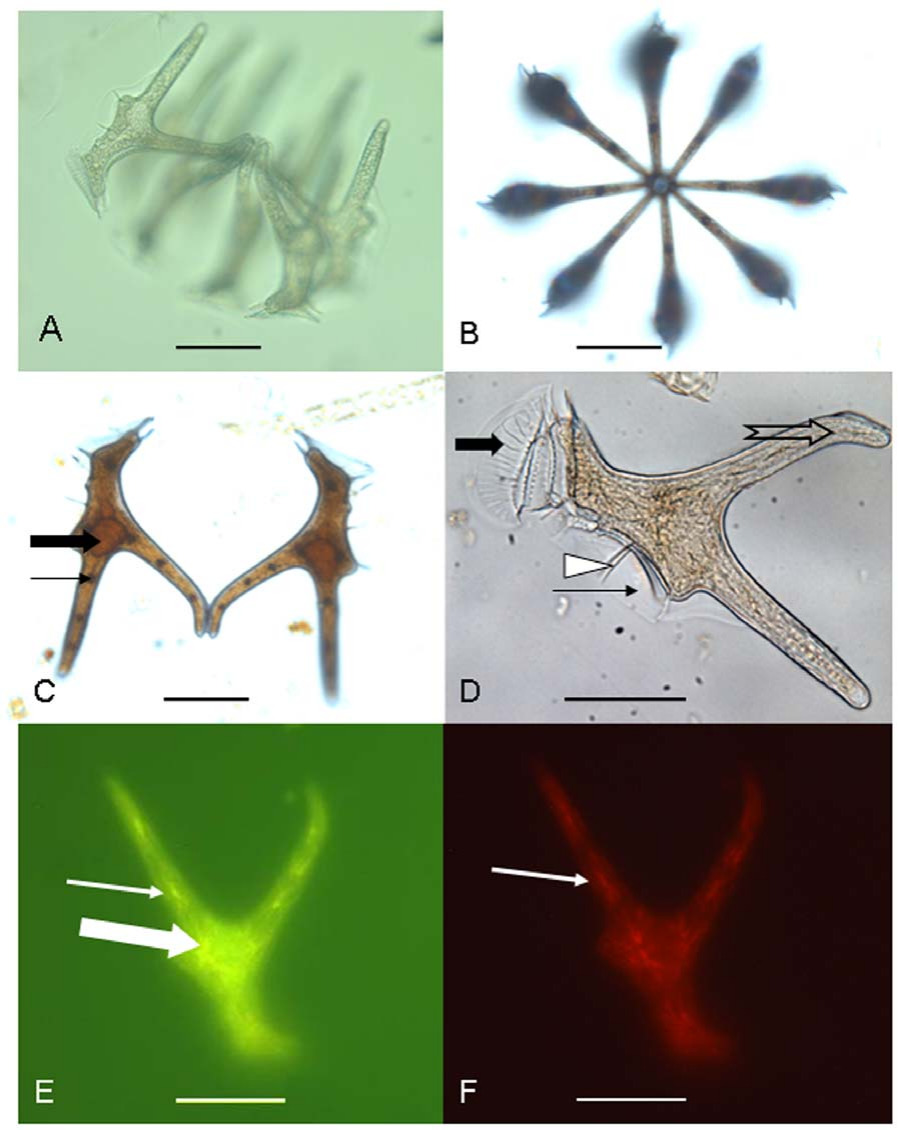
Micrographs of *Dinophysis miles* collected in this study. a) Side view of a 8-cell colony. b) Apical view of the 8-cell colony. c) Close-up view of two cells to show their attachment to each other at the end of the dorsal process, the visible nucleus (thick arrow), and the dark-stained plastid by Lugol's indicative of starch storage (thin arrow). d) A cell after Lugo's stain was removed, showing the anterior list (thick arrow), the sulcal list (thin arrow), and ribs (dashed arrow). e) Green fluorescence under blue light excitation of DNA stained with SYBR Green I in the nucleus (thick arrow) and plastid (thin arrow). f) Orange fluorescence from phycoerythrin in the plastids (arrow) under green excitation light. Scale bar = 50 µm in Fig. 1 A–F.

Microscopic examination confirmed that the isolated cells (Fig. 1) were morphologically identical to *D. miles* var. *indica*. The cells had two posterior projections that extended from the end of the hypotheca, which are characteristic of *D. miles* and *D. tripos*. In contrast to *D. tripos*, our sorted cells had slim cell bodies and the dorsal process was longer than that of *D. tripos*, plus the ends of the processes were smooth, which is typical of *D. miles*. The angle between the two projections was about 70°, matching that of *D. miles* var. *indica* [32]. The cell concentration ranged from 28 to 34 cells L^−1^. The size of *D. miles* cell was about 16–21 µm in width and 140–165 µm in length. Most of the cells were found in eight-cell colonies (Fig. 1A, B) except two-cell pairs in some cases (Fig. 1C). The eight cells formed a ring by attaching to each other at the end of the dorsal process of the cell (Fig. 1C), i.e. the process opposite to the sulcal list (Fig. 1D). In the cells of *D. miles* that were examined under the microscope, 5–10 plastids-like entities (n = 10) were observed, which showed dark staining of starch deposit by Lugol's solution (Fig. 1D), indicating plastids likely of cryptophyte origin. After removal of Lugol's stain followed by DNA staining using SYBR Green I, DNA fluorescence (Fig. 1E) and pigment autofluorescence (Fig. 1F) were apparent under the epifluorescence microscope.

### Phylogentic position of *D. miles* based on nuclear rDNA and mitochondrial *cob* and *cox1*

**Figure 2:**
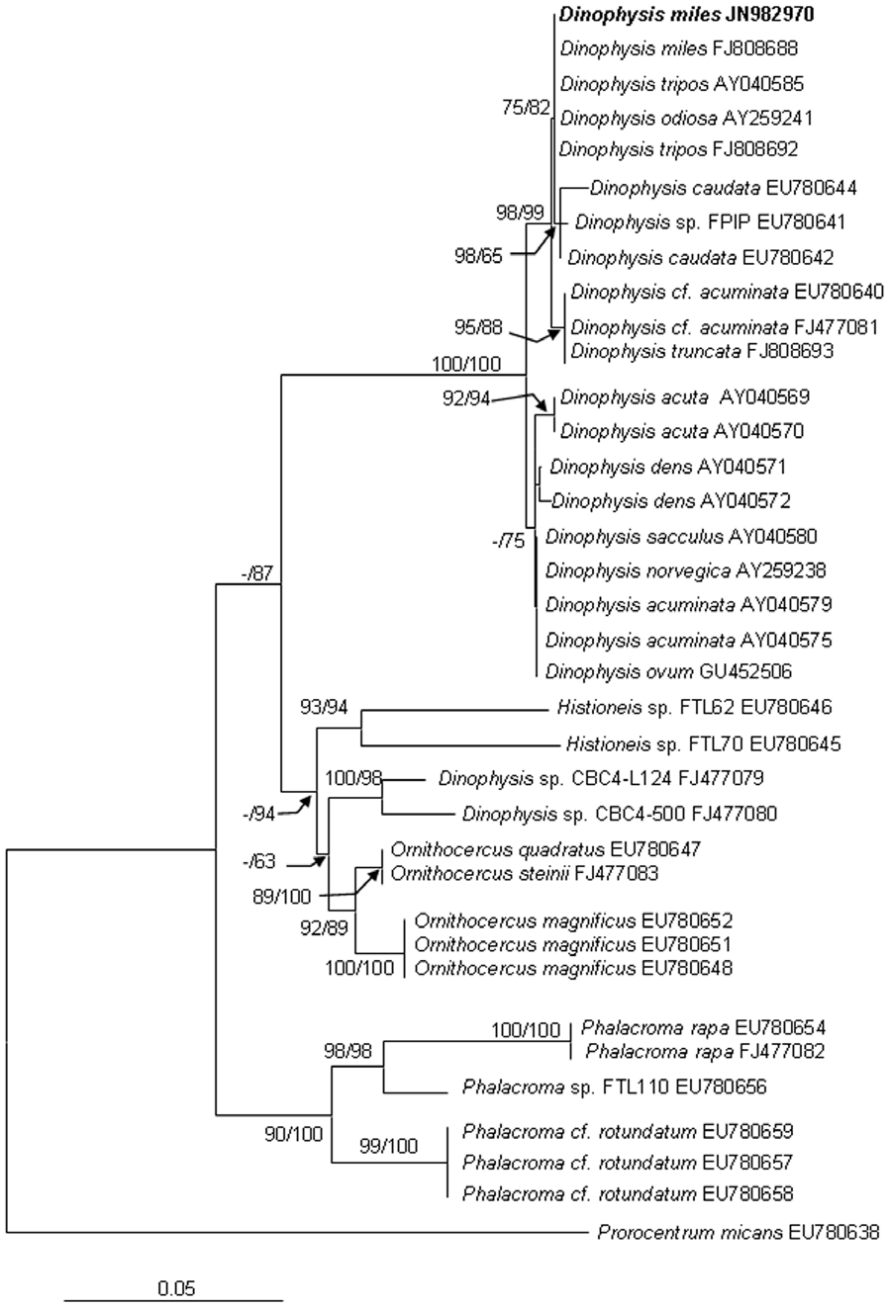
Phylogenetic relationship of *D. miles* with other dinophysioid dinoflagellates inferred from LSU rDNA. Sequence obtained in this study is bold-typed. Support of nodes is based on bootstrap values of ML/NJ with 1000 and 500 resamplings, respectively. Only values greater than 60 are shown. If only one of the two phylogenetic methods yielded significant support, the other is shown with “-”. *Prorocentrum micans* was used as the outgroup to root the tree. In this tree, *D. miles* cannot be separated from *D. tripos* and *D. odiosa*.

**Figure 3:**
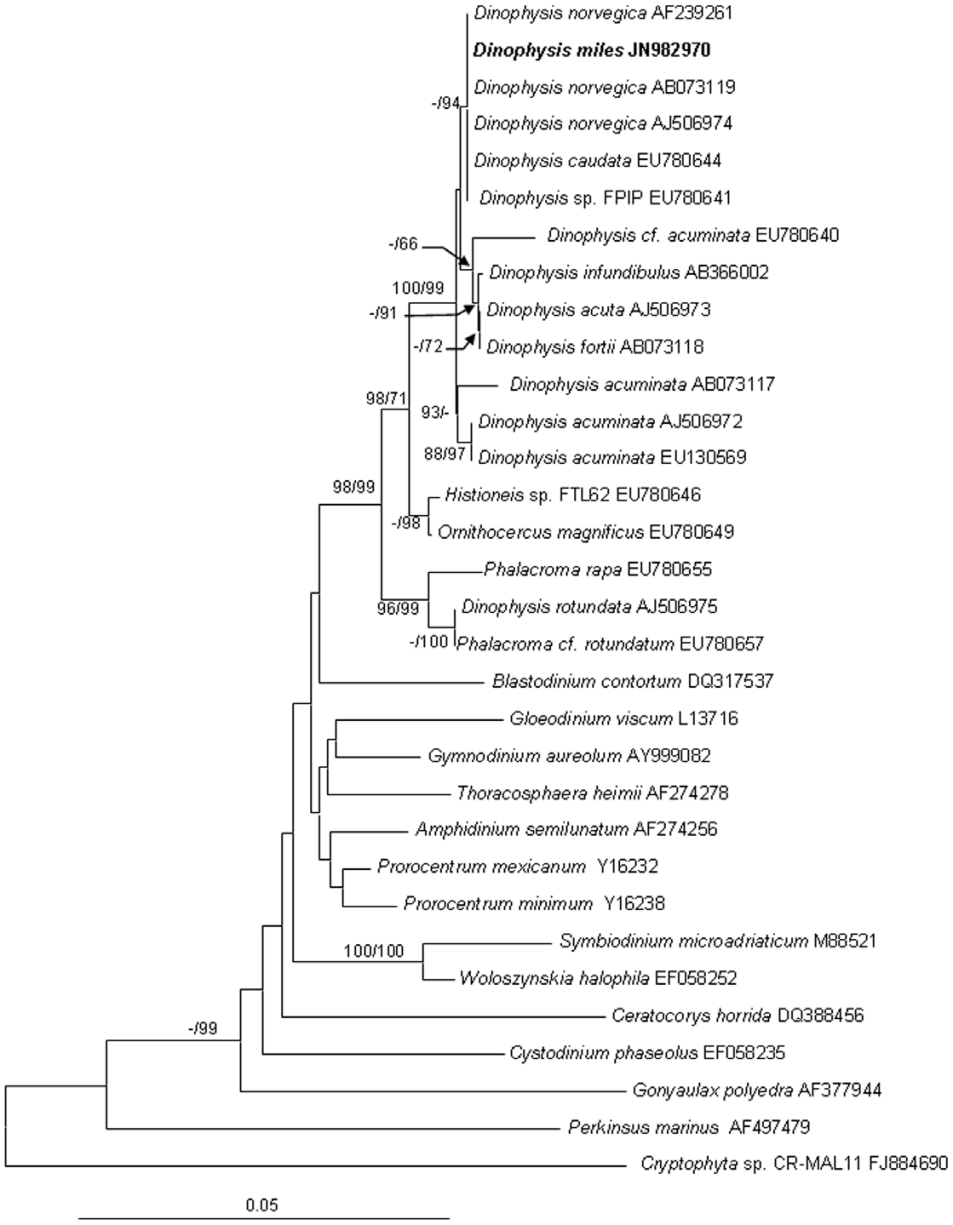
Phylogenetic relationship of *D. miles* with other dinoflagellates inferred from SSU rDNA. Sequence obtained in this study is bold-typed. Support of nodes is based on bootstrap values of ML/NJ with 1000 and 500 resamplings, respectively. Only values greater than 60 are shown. If only one of the two phylogenetic methods yielded significant support, the other is shown with “-”. *Cryptophyta* sp. was used as the outgroup to root the tree. In this tree, *D. miles* cannot be separated from *D. norvegica* and *D. caudata*.

**Figure 4:**
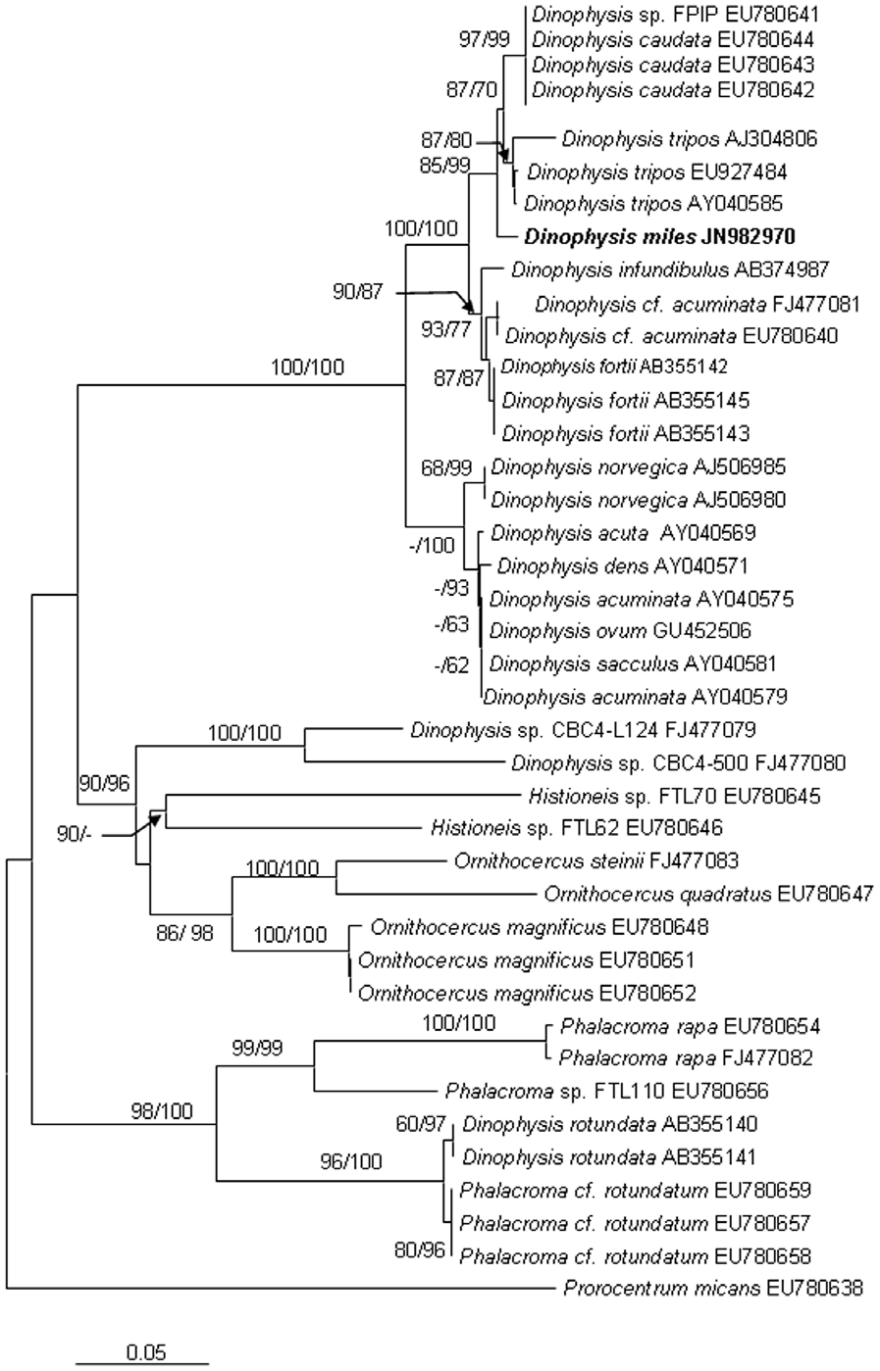
Phylogenetic relationship of *D. miles* with other dinophysioid dinoflagellates inferred from ITS1-5.8S-ITS2. Sequence obtained in this study is bold-typed. Support of nodes is based on bootstrap values of ML/NJ with 1000 and 500 resamplings, respectively. Only values greater than 60 are shown. If only one of the two phylogenetic methods yielded significant support, the other is shown with “-”. *Prorocentrum micans* was used as the outgroup to root the tree. In this tree, *D. miles* appears as a distinct lineage, well separated from *D. tripos*, *D. norvegica*, *D. caudata*, and other *Dinophysis* species.

We obtained the nuclear-encoded ribosomal RNA sequence 2,824-bp (JN982970) from the sorted cells, composed of the partial sequence of SSU, ITS1, 5.8S, ITS2, and the partial sequence of LSU (D1–D2). Within the 2.824-kb sequence, the dinoflagellate SSU region spanned 1.59 kb (nucleotide positions 1–1593), the ITS1-5.8S-ITS2 region (abbreviated as ITS hereafter) 0.59 kb (positions 1557–2146), and the LSU region 0.68 kb (positions 2147–2824). The phylogenetic tree of SSU, ITS and LSU included 32, 40 and 36 sequences, respectively from Genbank, in addition to the sequences obtained in this study. The topologies of these trees inferred from the three datasets using Neighbor Joining (NJ) and Maximum Likelihood (ML) were similar and indicated clear separation of well-supported four genera, *Phalacroma*, *Histioneis*, *Ornithocercus* and *Dinophysis* (Figs. 2, 3, 4). In all three sets of trees, the genus of *Dinophysis* (such as *D. acuminata* and *D. acuta*) was distinct from other species. However, resolution of *D. miles* from other *Dinophysis* species varied among the three genes. In the LSU tree (Fig. 2), the South China Sea *D. miles* was identical to a sequence reported for *D. miles* from the Indian Ocean (FJ808688), but appeared to be identical also to *D. tripos* (FJ808692, AY040585) and *D. odiosa* (AY259241). Thus LSU was unable to resolve the three species. In the SSU tree (Fig. 3), *D. miles* could not be separated from *D. caudata* (EU780644) and *D. norvegica* (AF239261, AB073119, AJ506974). In contrast, ITS phylogeny placed *D. miles* as a distinct lineage, well separated from *D. caudata* (EU780642, EU780643, EU780644), *D. tripos* (AJ304806, EU927484, AY040585), and other *Dinophysis* species (Fig. 4). LSU and ITS results combined verified the morphological identification of the sorted cells as *D. miles*. Based on all the three sets of trees, *D. miles* appeared to be closely related to *D. tripos* and *D. caudata*.

**Figure 5:**
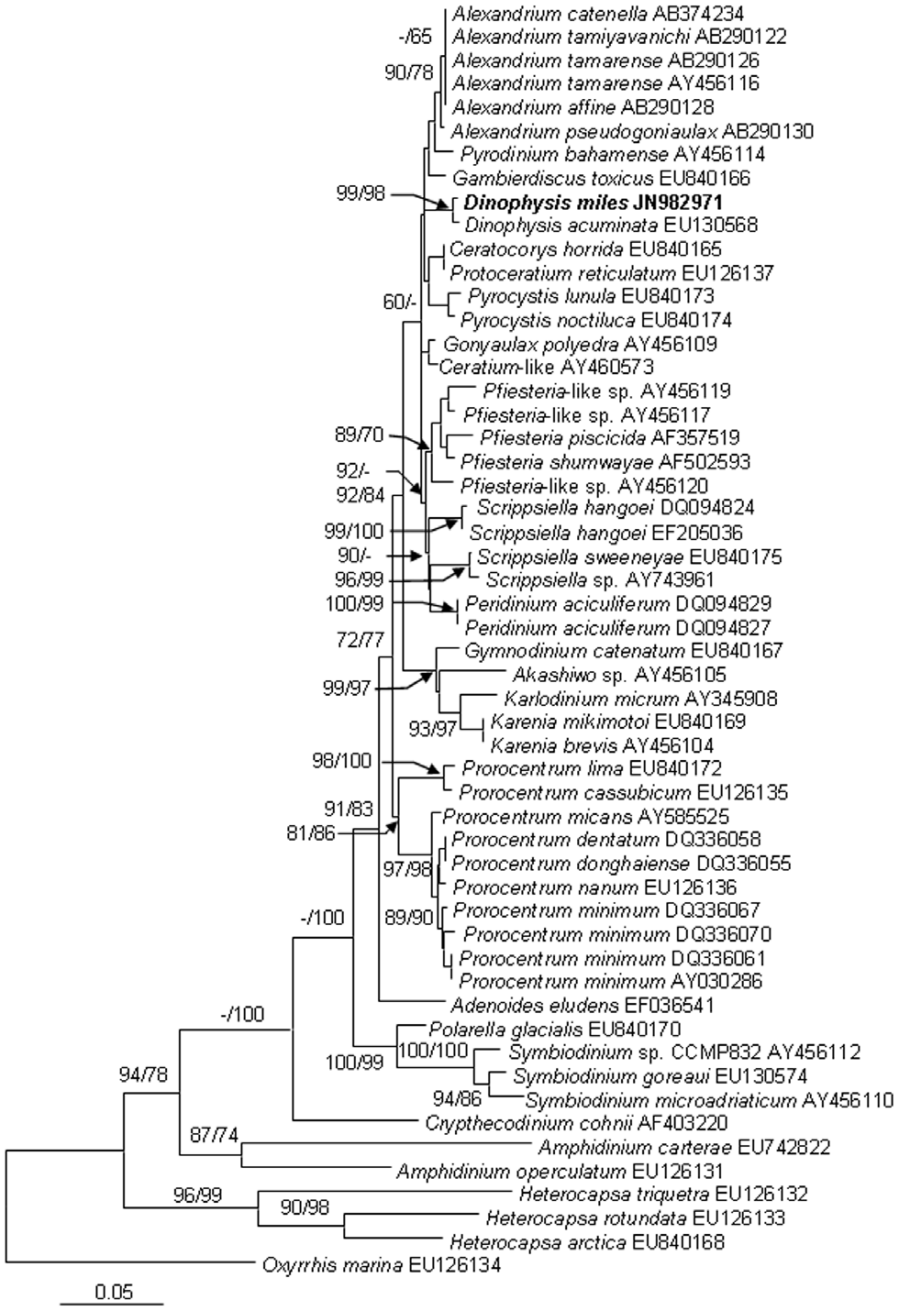
Phylogenetic relationship of *D. miles* with other dinoflagellates inferred from *cob*. Sequence obtained in this study is bold-typed. Support of nodes is based on bootstrap values of NJ/ML with 1000 and 500 resamplings, respectively. Only values greater than 60 are shown. If only one of the two phylogenetic methods yielded significant support, the other is shown with “-”. *Oxyrrhis marina* was used as the outgroup to root the tree. In this tree, *D. miles* is separated from *D. acuminata*, the only *Dinophysis* species whose *cob* sequence is available.

**Figure 6:**
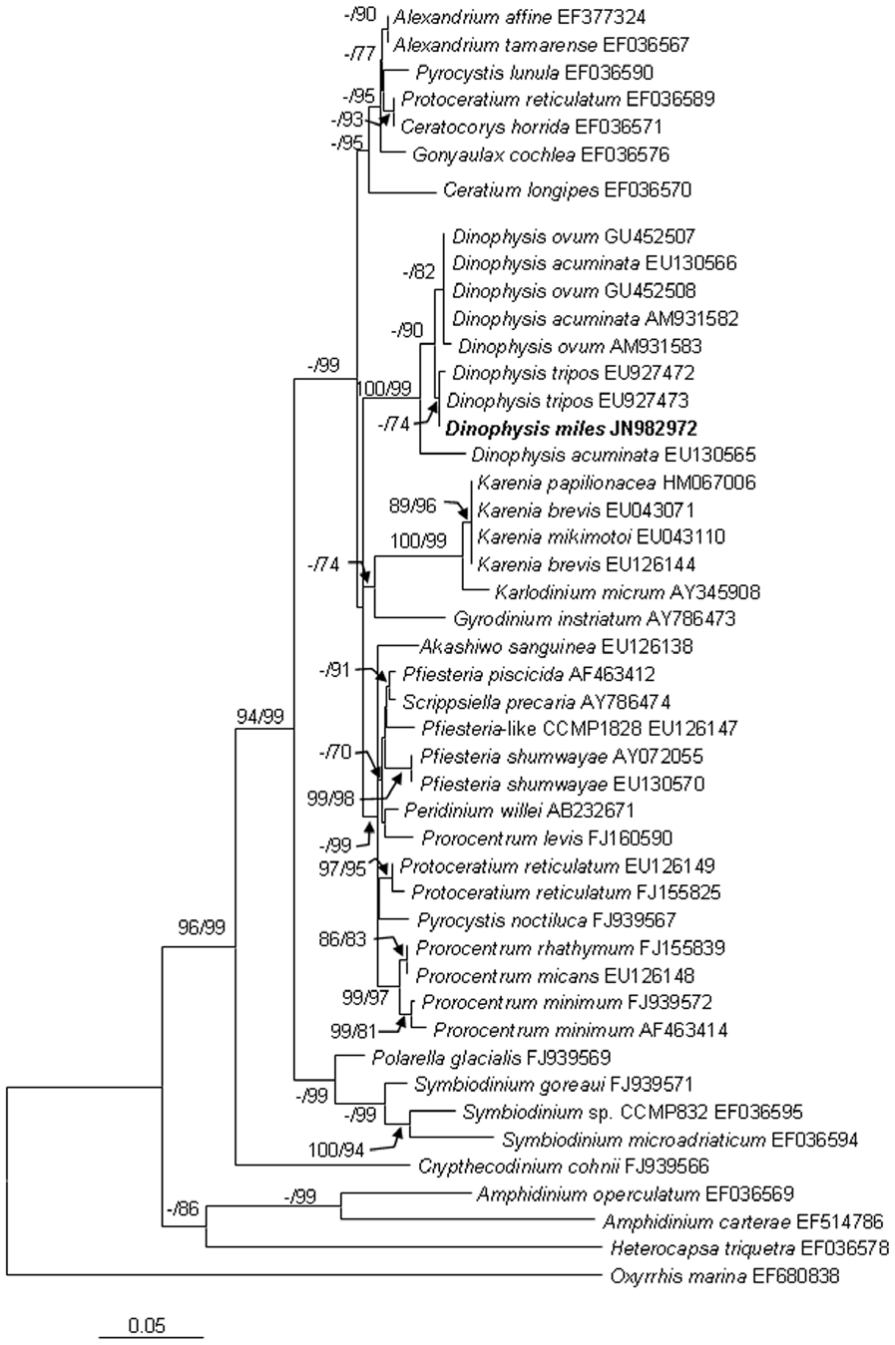
Phylogenetic relationship of *D. miles* with other dinoflagellates inferred from *cox*1. Sequence obtained in this study is bold-typed. Support of nodes is based on bootstrap values of NJ/ML with 1000 and 500 resamplings, respectively. Only values greater than 60 are shown. If only one of the two phylogenetic methods yielded significant support, the other is shown with “-”. *Oxyrrhis marina* was used as the outgroup to root the tree. In this tree, *D. miles* is separated from *D. ovum* and *D. acuminata*.

The alignment of *cob* consisted of the *D. miles* sequence obtained (JN982971) in the present study and 55 sequences from other dinoflagellates available in GenBank. The 913-bp *cob* sequence from *D. miles* var. *indica* differed by only 3 bp (0.33%) from that of *D. acuminata* (EU130568), the only *Dinophysis cob* sequence available in GenBank. The *cox*1 sequence obtained from *D. miles* var. *indica* (JN982972, 840-bp) contained the widely used DNA barcode region (∼650-bp) [40]. It was aligned with 46 homologous sequences from other dinoflagellates available in GenBank. The *cox*1 sequences from *D. miles* var. *indica* differed by only 3 or 4 bp (0.36% or 0.48%) from counterparts of *D. ovum* (AM931583, GU452507, GU452508), and also only 3 bp (0.36%) from a *D. acuminata* sequence (EU130566, mRNA sequence is EU130565), and 0 bp or only 1 bp (0.24%) from *D. tripos* sequences (EU927473, EU927472). *Cob* and *cox*1 molecular phylogenies showed that *Dinophysis* species formed strongly supported lineages (Fig. 5, 6).

### Phylotypes of the plastid

**Figure 7:**
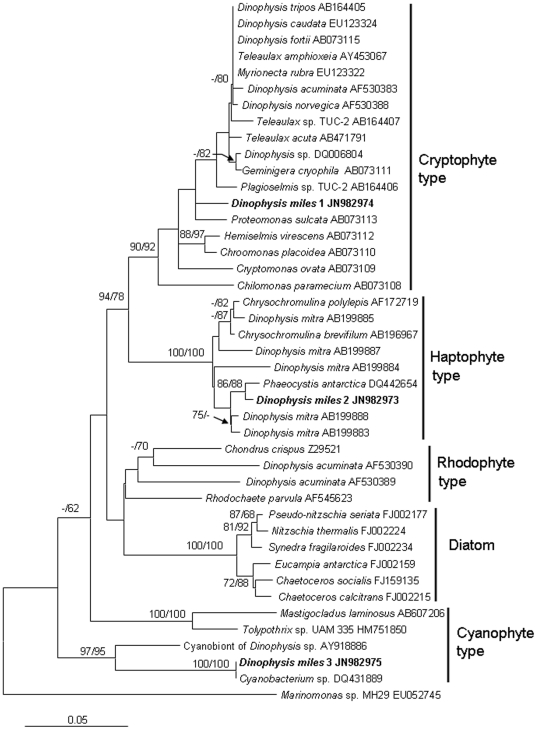
Phylogram of plastid SSU rDNA showing diverse types of plastids and symbionts in *D. miles*. Sequence obtained in this study is bold-typed. Support of nodes is based on bootstrap values of NJ/ML with 1000 and 500 resamplings, respectively. Only values greater than 60 are shown. If only one of the two phylogenetic methods yielded significant support, the other is shown with “-”. *Marinomonas* sp. was used as the outgroup to root the tree.

Sequencing results revealed three types of plastid SSU rDNA sequences (JN982973–JN982975) from colonies of *D. miles* var. *indica*. BLAST analyses of the 423-bp sequences indicated that they belonged to different lineages. One (JN982974) was 96% identical to the plastid SSU of the cryptophytes *Teleaulax amphioxeia* (AY453067) and *Plagioselmis* sp. TUC-2 (AB164407), one (JN982973) 98% identical to that of the haptophyte *Phaeocystis antarctica* (DQ442654) and the plastid SSU of *D. mitra* (AB199888), and the other (JN982975) 100% identical to that of an uncultured cyanobacterium (DQ431889) and 91% identical to that of the cyanobionts of *Dinophysis* sp. (AY918886). Phylogenetic analyses also showed that these *D. miles* var. *indica* sequences clustered with the plastid SSU of cryptophytes, haptophytes and cyanophytes, respectively (Fig. 7). Of these, the cryptophytes-type clade comprises cryptophytes and the majority of photosynthetic *Dinophysis* species; the haptophyte-type clade consists of haptophytes and several populations of *D. mitra*; the rhodophyte-type clade contains rhodophytes and *D. acuminata*; the cyanophyte-type clade is composed of cyanobacteria and *Dinophysis* sp.. While *D. acuminata* is represented in two (cryptophyte and rhodophyte) clades, only *D. miles* var. *indica* covers three clades.

## Discussion

Analyzing natural populations of a dinoflagellate species alleviates the barrier of lack of cultures to study the species. The culture-independent approach also is the only way to gain understanding on physiological and molecular genetic characteristics in the natural populations. As the first study dedicated to *D. miles*, we have sequenced SSU and ITS in *D. miles* var. *indica*, and analyzed *Dinophysis* phylogenies based on nuclear SSU-ITS-LSU and mitochondrial *cob* and *cox*1 to compare their performance in distinguishing different species within this genus. The sequences obtained and the results of phylogenetic analyses will be useful for future phylogenetic and DNA barcoding studies for this and related species. Further, analysis of plastid SSU on the natural population of *D. miles* reveals multiple plastids (and cyanobionts) associated with this species, a finding that would be difficult to obtain using laboratory cultures. Therefore, taking advantage of culture-independent molecular techniques, research on natural populations of dinoflagellates has the potential of yielding more information. This potentially can be applied to other protists that are amenable to single cell (colony) isolation, which is becoming increasingly feasible with the aid of flow cytometry (e.g., [41]). However, working directly on natural populations of protists is challenging because it is often difficult to isolate the target species from the plankton assemblage and it is prone to contamination by co-existing organisms. In our study, *D. miles* is relatively large in cell size, and hence relatively easy to isolate. Careful washing and microscopic examination further minimized the chance of contamination by other phytoplankton.

### Comparison of phylogenies based on the three regions in the nuclear rDNA sequences and mitochondrial *cob* and *cox1*

Morphological observations augmented by molecular analyses indicate that the *Dinophysis* population we detected was *D. miles* var. *indica*. Molecular phylogenies indicate that nuclear SSU, ITS, LSU rDNA and mitochondrial *cob* and *cox*1 all have sufficient resolving power to discriminate genera in Dinophysiales. Our results showed that among these gene regions, the ITS region offered the best resolution between *D. miles* and other *Dinophysis* species. The phylogenies of the nuclear rDNA regions showed varying interspecific distances in the genus of *Dinophysis*. LSU fails to differentiate the morphologically similar species *D. miles*, *D. tripos*, as well as the morphologically more distinct *D. odiosa*, and SSU could not distinguish *D. miles* from *D. norvegica* and *D. caudata*. Handy *et al*. (2009) indicated that the nuclear-encoded ITS1 and ITS2 have undergone higher evolutionary rate than LSU and SSU rDNA regions based on a comparison of percent identity among *Histioneis* sp., *Ornithocercus magnificus*, and *Dinophysis* spp. relative to *Phalacroma rapa* [4].

In the *cob* phylogenic tree, *D. miles* is closely related to, but different from, *D. acuminata* among other dinoflagellates. The sequence we obtained embraced a 334-bp region, which has been demonstrated to be a promising DNA barcoding marker for dinoflagellate species [40]. This gene sequence exhibit only three nucleotide difference between *D. miles* and *D. acuminata*, two of which are located within the 334-bp region. The separation of these two species is consistent with the result based on rRNA genes, but the overall resolving power of this gene for *Dinophysis* species remains to be determined in further studies with broader taxon sampling.

In the *cox*1 phylogenic tree, *D. miles* is well resolved from *D. acuminata* and *D. ovum* although their distances were short. *D. miles* and *D. ovum* only differed by 3 or 4 bp (0.36% or 0.48%). *D. miles* differed from a previously reported *D. acuminata* sequence (EU130566) by 3 bp (0.36%) yet from another (AM931582) by 9 bp (1.07%). These two reported *D. acuminata cox*1 sequences showed a difference of 93 bp (7.74%), which is unprecedented and highly unlikely for any dinoflagellates. Raho *et al.* (2008) based on their sequence of *D. acuminata* (AM931582) concluded that the *cox*1 region had higher resolving power than ITS [42]. Our results show that this is not the case, casting question on the accuracy of that reported sequence. Careful comparison of AM931582 with EU130566 and counterpart sequences from other *Dinophysis* species showed that the apparent variable sites in AM931582 were mostly in the 3′ end, suggesting possibility of sequencing errors toward the end of read length. Alternatively, host of the AM931582 might have been a totally unrelated organism misindentified as *D. acuminata*. Furthermore, previously reported *cox*1 sequence from *D. tripos* (EU927473) was identical to the *D. miles* sequence (JN982971) obtained in this study. Unlikely, this gene would separate the two species so well.

Because ITS as a non-coding region has higher variability than the coding regions SSU and LSU, it is expected to have greater resolving power for all eukaryotes. The usefulness of ITS in resolving dinoflagellate species has been demonstrated [43]. Consistent with these findings, our results also showed that the ITS region separated *D. miles* from *D. tripos*, *D. acuminata*, and other *Dinophysis* species with strong bootstrap support (Fig. 4), indicating its greater resolving power for *D. miles* and related species. In contrast, as shown above, the SSU, LSU, and the two mitochondrial genes, overall show lower, albeit varying, levels of resolving power between *Dinophysis* species. Therefore, ITS1-5.8S-ITS2 region seems to be the most effective region to distinguish *D. miles* from other *Dinophysis* species among these five gene loci. In addition, based on all the current phylogenies inferred from the five gene loci, *D. miles* is closely related to *D. tripos* and *D. caudata* and more distant from *D. acuminata*.

### “Plastid” consortium in *D. miles*

In this study, we retrieved three different types of plastid SSU rDNA sequences from *D. miles* var. *indica*. Based on the phylogenetic analyses of the plastid genes, two plastid sequences are of crytophyte and haptophyte origin, the third sequence is closely related to cyanobacterial SSU. These different plastid SSU sequences are unlikely to be a result of contamination. First, microscopic examination of our net tow samples showed predominance of diatoms (*Chaetoceros*, *Rhizosolenia* and other genera); any cryptophytes, haptophytes, or cyanobacteria cells present in the study ocean area would have been mostly lost through the 55-µm mesh during the net tow. Second, our picked cell colonies were extensively rinsed in filtered seawater before DNA extraction. Furthermore, cryptophyte and haptophyte plastids have both been demonstrated to be plastids in *Dinophysis* spp. and cyanobacteria have been reported to associate with some dinophysioids. Our microscopic observation on some of the cells we isolated revealed the intracellular plastid stained intensely with iodide, indicative of starch storage, and phycoerythrin-like fluorescence, indicating presence of cryptophyte type of plastid or cyanobacteria inside *D. miles* var. *indica* cells. Therefore, the *D. miles* var. *indica* population in the South China Sea likely possesses a consortium of plastids and cyanobionts previously documented separately in different dinophysioids species.

One of the plastid SSU sequences retrieved in our study is most closely related to that in *Proteomonas sulcata*. One the one hand, this agrees with the previous results that most of the *Dinophysis* species contain plastids originated from cryptophytes [16], [18], [22] (Table 2); on the other hand, this distinguishes *D. miles* from most of *Dinophysis* spp. which have plastids originating from a different cryptophyte [15], [16], [19]. The second plastid SSU sequence found from *D. miles* var. *indica* is of haptophyte origin, similar to *D. mitra* from Okkirai Bay, Japan [23] (Table 2). Intriguingly, the *D. mitra* population harbors plastids of different haptophyte lineages, including those closely related to *Phaeocystis* and *Chrysochromulina*, respectively, suggesting that these are kleptoplastids retained from prey algae, in contrast to the more controversial status of cryptophyte-derived plastids in other *Dinophysis* species. The haptophyte-type plastid of *D. miles* var. *indica* is most closely related to plastids of *Phaeocystis antarctica* (Fig. 7). Interestingly, Gast *et al*. (2007) showed that a haptophyte alga closely related to *Phaeocystis antarctica* was grazed by a dinoflagellate in the Ross Sea, Antarctica, and its plastid was retained in the dinoflagellate cell for temporary photosynthesis [44]. This suggests that grazing and retention of haptophyte plastids by dinoflagellates occur in both polar and tropical waters, and are likely a widespread phenomenon in dinoflagellates.

**Table 2:** Types of plastids found in *Dinophysis* spp.

Source	Study sites	Cryptophyte origin	Rhodophyte origin	Haptophyte origin	References
*D. norvegica*	Baltic Sea; Okkirai Bay and Funka Bay, Japan; Clam Cove, Maine; Masfjord and North Sea	Y			Carpenter *et al.*, 1995; Takahashi *et al.*, 2002, 2005; Hackett *et al.*, 2003; Minnhagen and Janson, 2005
*D. tripos*	Okkirai Bay and Funka Bay, Japan	Y			Takahashi *et al.*, 2005; Nishitani *et al.*, 2010
*D. caudata*	Near Namhae, Korea; Yatsushiro Sea, Japan	Y			Park *et al.*, 2008; Nishitani *et al.*, 2010
*D. infundibulus*	Funka Bay, Japan	Y			Nishitani *et al.*,2010
*D. fortii*	Okkirai Bay, Hiroshima Bay, Yatsushiro Sea and Notoro saline lake, Japan	Y			Takahashi *et al.*, 2002, 2005; Nishitani *et al.*, 2010
*D. acuta*	Ninigret Pond, USA	Y			Hackett *et al.*, 2003
*D. acuminata*	Kesennuma Bay, Yatsushiro Sea, Funka Bay and Okkirai Bay, Japan; Greenwich Cove and Watch Hill Cove, Rhode Island; Near Frederikssund, Denmark; Masfjord and Baltic Sea	Y			Takishita et al., 2002; Hackett *et al.*, 2003; Nishitani *et al.*, 2010; Garcia-Cuetos *et al.*, 2010; Minnhagen and Janson, 2005
*D. acuminata*	Greenwich Cove, Rhode Island	Y	Y		Hackett *et al.*, 2003
*D. mitra*	Okkirai Bay, Japan			Y	Koike *et al.*, 2005
*D. miles*	South China Sea	Y		Y	This study

The third plastid-like SSU sequence from *D. miles* var. *indica* belongs to the lineage of cyanobacteria. While cyanobacteria have been shown to be endosymbionts of some dinophysioid species [12]–[14], most cyanobacterial associations are believed to behave as extracellular symbionts (cyanobionts). Cyanobionts occur in three genera of Dinophysiaceae, *Citharistes*, *Histioneis*, and *Ornithocercus* and our finding extends that to the genus of *Dinophysis* [4], [8]–[10]. It was thought that the lists that develop from extended cingulum and sulcus provide a habitat for the cyanobionts in some dinophysioids [4], [45]–[47]. *Histioneis* and *Ornithocercus* possess prominent lists on the epicone or cingulum for the ectophytic cyanobionts to reside in [13]. It was postulated that in *Phalacroma* and *Dinophysis* both the cingular and sulcal lists are not so elaborate and as a result no cyanobionts occur on them [4], [46], [47]. It is unclear if the cyanobionts detected in *D. miles* are endosymbiotic or ectosymbiotic. Our microscopic observations showed that *D. miles* cells had a well-developed anterior cingular list, sulcal list and rib systems (Fig. 1 C, D), suggesting that it is suited for cyanobionts to inhabit. Handy *et al.* (2009) showed, based on SSU phylogeny, that *Histioneis* and *Ornithocercus* cluster together and both have cyanobionts; in contrast, *Dinophysis* and *Phalacroma* were separated from those two genera and did not have cyanobionts [4]. However, in our nuclear SSU, ITS, and LSU phylogenetic trees, *Dinophysis*, *Histioneis*, and *Ornithocercus* consistently clustered together, and the clade was distinct from *Phalacroma*. *Citharistes* was not included in our analyses due to the unavailability of SSU and ITS sequences and its phylogenetic relationship with those lineages could not be confirmed. Nevertheless, our nuclear rDNA phylogenetic analysis results consistently show that *Dinophysis* as well as *Histoneis* and *Orthithocercus* can host cyanobionts. It is noteworthy that our detected cyanobacterial sequence is 91% identical to recently reported cyanobionts of *Dinophysis* sp. cells [48].

Three plastid-types suggest a possibility that *D. miles* has cryptic species that acquire different types of plastids. They can also be indication that *Dinophysis* nutritional physiology is more complicated than currently understood. The cryptophyte-type plastid seems to be the most common among *Dinophysis* spp., although whether it is a permanent or temporary (kleptoplastid) plastid is still being debated [17]–[19], [21]. The only exception is in *D. mitra*, if verified by further research. The different type of cryptophytes found in *D. miles* var. indica suggest that the cryptophyte plastid is probably not a permanent and universal plastid for the genus of *Dinophysis*. The failure to detect plastid-maintaining gene transcripts in *D. acuminata* [21] further supports the case for kleptoplastidy. The more variable and spotty presence of haptophyte (*D. mitra*, *D. miles*), rhodophyte (*D. acuminata*, Table 2), and cyanobacteria (*Dinophysis* sp., *D. miles*) most likely indicate the availability and the selection (if any) in the environment by the different *Dinophysis* species. This remains a question that can be answered only by systematic investigation on *Dinophysis* species and their sympatric phytoplankton assemblages in the natural environments. Further studies are also needed to determine whether all these photosynthetic entities are present in every single *D. miles* cell in the population, and whether they all are functional for photosynthesis and benefit the growth of the *D. miles* var. *indica* population.
